# Biodegradable Carbon-based Ashes/Maize Starch Composite Films for Agricultural Applications

**DOI:** 10.3390/polym12030524

**Published:** 2020-03-01

**Authors:** Enrica Stasi, Antonella Giuri, Francesca Ferrari, Vincenza Armenise, Silvia Colella, Andrea Listorti, Aurora Rizzo, Eleonora Ferraris, Carola Esposito Corcione

**Affiliations:** 1 Dipartimento di Ingegneria dell’Innovazione, Università del Salento, 73100 Lecce, Italy; enrica.stasi@unisalento.it (E.S.); francesca.ferrari@unisalento.it (F.F.); 2 Istituto di Nanotecnologia CNR-Nanotec c/o Campus Ecotekne, Via Monteroni, 73100 Lecce, Italy; antonella.giuri@unisalento.it (A.G.); andrea.listorti@uniba.it (A.L.); aurora.rizzo@unisalento.it (A.R.); 3 Dipartimento di Chimica, Università di Bari “A. Moro”, via Orabona, 4, 70126 Bari, Italy; vincenza.armenise@uniba.it; 4 Istituto di Nanotecnologia CNR-Nanotec c/o Dipartimento di Chimica, Università di Bari “A. Moro”, via Orabona, 4, 70126 Bari, Italy; silvia.colella@unisalento.it; 5 Department of Mechanical Engineering, Campus de Nayer, 2860 KU Leuven, Belgium; eleonora.ferraris@kuleuven.be

**Keywords:** carbon based ashes, maize starch, thermoplastic films, thermo-mechanical characterization, agricultural application

## Abstract

The aim of this work is the development and characterization of biodegradable thermoplastic recycled carbon ashes/maize starch (TPAS) composite films for agricultural applications. A proper plasticizer, that is, glycerol, was added to a commercial maize starch in an amount of 35 wt.%. Carbon-based ashes were produced by the biomass pyro-gasification plant CMD ECO 20, starting from lignocellulosic wastes. The ashes were added to glycerol and maize native starch at different amounts ranging from 7 wt.% to 21 wt.%. The composite was mixed at 130 °C for 10 min and then molded. The effect of the different amounts of carbon based ashes on the thermal and physical-mechanical properties of the composite was assessed by using several techniques, such as rheology, wide-angle X-ray diffraction (WAXD), scanning electron microscopy (SEM), differential scanning calorimetry (DSC), thermogravimetric analysis (TGA), moisture absorption, degradation and mechanical tests. The presence of the carbon waste ashes allows to improve thermal and durability performances of the thermoplastic starch (TPS) films. It reduces the water absorption of starch matrix and strongly decreases the deterioration of starch, independently from fillers amount, enhancing the lifetime of the TPS films in outdoor conditions. In addition, the waste carbon ashes/maize starch films present an advantage in comparison to those of neat starch; it can biodegrade, releasing the plant nutrients contained in the ashes into the soil. In conclusion, this approach for recycling carbon waste ashes increases the efficiency of industrial waste management, along with a reduction of its impact on the environment.

## 1. Introduction

This study suggests a novel and cost-effective reutilization of carbon waste ashes as reinforcing fillers of biocomposite films, based on thermoplastic starch, for agricultural applications. To the best of our knowledge this approach was never been proposed before, especially in the field of biodegradable covers of agricultural crops, covers that are able to release nutrients into the soil. Currently, the employment of carbon-based ashes mostly includes: (i) the improvement and fertilization of forest and agricultural soils; (ii) the production of construction materials (such as cement and concrete); (iii) new effective adsorbents; (iv) the synthesis and production of minerals, ceramics and other materials [1]. On the other hand, several studies have been already performed on the re-use of different waste materials as fillers for thermoplastic biopolymers, such as polylactic acid (PLA), Poly(3-hydroxy-butyrate) or starch. Lactic acid (LA), which is produced by chemical conversion of corn or other carbohydrate sources into dextrose, is a biodegradable, bio-based (bio-derived) and renewable polymer. Despite being a promising biopolymer, with good mechanical properties, PLA has a higher cost of production if compared to petroleum-derived polymers. Also, another drawback lies in its high brittleness [2]; nevertheless, several studies demonstrated that natural plasticizers can be used to improve the toughness of PLA [3,4]. Poly(3-hydroxy-butyrate) (PHB) is obtained by the Alcaligens eutrophorus bacteria; as it is produced by the bacterial fermentation of renewable resources, PHB is considered one of the most interesting biodegradable polymers. Moreover, PHB shows a hydrophobic behavior and, being a linear saturated polyester, can be easily processed at relatively low temperatures, thus obtaining mechanical performances comparable to those of PP. On the other hand, PHB has the disadvantage of being thermal instable when processed to temperatures closed to the melting point, showing a relatively low impact resistance [5,6]. Noticeably, starch is abundant, cost effective, sustainable and biodegradable. It has a semicrystalline structure, composed of 1,4-linked α-d-glucopyranosyl units with the two major macromolecular chains being the amylose and amylopectin [7]. The amylose is an almost linear polymer with α-d-(1,4) glycosidic linkages, while the amylopectin is a highly branched polymer which also contains α-d-(1,6) glycosidic linkages at the branching points, in addition to α-d-(1,4) glycosidic linkages [8]. Starch is one of most commonly used biopolymer in many application fields, such as in agriculture, food and pharmaceutical industries for the synthesis of films and coatings [7]. 

The interest in using thermoplastic starch in agricultural applications initiated in the 1970s and intensified in the 1980s, along with the dramatic growth in the utilization of plastics worldwide and the concerns on its effects on the environment. In the last years, several studies have specifically proposed the employment of thermoplastic starch-based composites as agricultural mulch films [9]. For instance, blends of starch and synthetic polymers, such as EVA and PVA, were investigated and resulted to be promising as films for paper or agricultural mulch [10]. In References [11,12], blends of starch and poly(ethylene-co-acrylic acid) were also processed into biodegradable films and earmarked as agricultural mulch or packaging material. Moreover, films composed of polybutyrate adipate terephthalate and starch were studied in Reference [13], to minimize soil temperature and humidity while increasing photosynthesis and peanut yield. In particular, the films with a starch amount of 150 g per kg presented similar results to the typically used synthetic polymer films, for the entire growing season. Furthermore, starch foils and poly(butylene adipate-co-terephthalate) blends were developed as cover films in the production of strawberries [14]. After four weeks, the ripe fruits were collected and the mean fresh weights (grams per fruit) of strawberries produced with commercial and biodegradable covering films were compared with each other and they did not vary significantly. However, after five weeks, the biodegradable film exhibited small cracks and reduced tensile strength and within 8 weeks the mechanical properties of the film were fully compromise—the weight decreased due to variations in temperature, humidity and solar radiation, which led to its biodegradation, crosslinking and photo degradation. On the contrary, in Reference [15], a biodegradable liquid, composed of oxidized maize starch and gelatin from leather residues, was utilized as coating of agricultural foils in the growth of rapeseed. The coated films demonstrated to be suitable for agricultural production and the rate of survival and yield of rapeseed increased. Finally, native and oxidized thermoplastic corn starch, with and without the addition of natural and modified bentonite (incorporating an eco-friendly natural polymer, such as chitosan) were proposed as soil cover [16]. The oxidized film evidenced higher degree of crystallinity and less plasticization than the native ones, due to the higher *T_g_*.

However, starch is sensitive to moisture and weak in terms of mechanical property [7]. Hence, it is typically combined with a number of fillers, even waste based fillers, to produce biocomposites with enhanced mechanical and physical properties. As an example, Benito-González I. et al. incorporated cellulose fillers (from Posidonia waste biomass) into thermoplastic corn starch films and observed significant improvements in the mechanical and water resistance performance [17]. In Reference [18], Ibrahim M.I.J. et al. prepared biodegradable composite films by using thermoplastic corn starch as matrix and corn husk fibers (which refers to the leaves covering the corn ear) as a reinforcing filler, evidencing a noticeable reduction in density and moisture content, less resistance to biodegradation and enhanced thermal stability of the composite films. Moreover, Collazo-Bigliardi S. et al. added hydrothermal extracts and cellulose fibers, obtained from coffee and rice husks, into corn starch films, conferring to the samples antioxidant and antibacterial characteristics and improved tensile properties [19]. Furthermore, Sreekumar P.A. et al. obtained microcrystalline cellulose from olive pits (waste material from the olive industry) and used them as a filler of thermoplastic corn starch films and these fillers enhanced the tensile properties [20]. Finally, according to [21], the incorporation of chestnut husks, a lignocellulosic agroforestry waste, in thermoplastic potato starch films can increase the elastic modulus of the samples. 

However, thermoplastic corn starch foils containing recycled carbon ashes have never been developed for agricultural applications. The use of recycled carbon ashes is not yet massively diffused and in a recent study an innovative reutilization of carbon-based ashes as catalysts and reinforcing filler for the crosslinking reaction of epoxy resins with amines was proposed [22,23]. It is expected, that these films in addition to covering crops have the advantage of biodegrading and releasing the plant nutrients of the ashes into the soil. The soil application of the carbon ashes, derived from the combustion of lignocellulosic biomass would ideally match the need of plants for nutrients and essential elements [1]. 

Therefore, this work deals with the use of carbon waste ashes as fillers of innovative biocomposite thermoplastic starch films for agricultural applications. The aim is to provide cultivation covering foils characterized by enhanced thermal and physical-mechanical properties, which can also biodegrade, meanwhile releasing the plant nutrients into the soil. Those carbon ashes are the waste of the pyro-gasification process of lignocellulosic biomass, carried out on the CMD ECO 20 plant, developed by Costruzioni Motori Diesel (CMD). Considering that those particles would be waste to be landfilled, their re-utilization would remove a considerable economic and environmental burden of the company. Hence, the here proposed recycling approach increases the efficiency of industrial waste management, along with a reduction of its negative effects on the environment.

## 2. Materials and Methods 

### 2.1. Materials

The maize starch Maizena used in this work was purchased from Unilever (Rome, Italy). The glycerol was supplied by Cruciani prodotti Crual s.r.l. (Rome, Italy) and it was used as a plasticizer. The ashes were produced by the biomass pyro-gasification plant CMD ECO 20. The company Costruzioni Motori Diesel (CMD, San Nicola la Strada, Italy) developed the CMD ECO 20 system to produce heat and electric power, starting from innovative lignocellulosic waste. 100 g of ashes were milled for 24 h in an aluminous porcelain jar (1.5 L), using alumina balls in ambient atmosphere. The mechanical milling was performed in a horizontal oscillatory mill MMS-Ball Mill, operating at ±25 Hz. 

Wide-angle X-ray diffraction (WAXD) patterns of the ashes were obtained using an automatic Bruker D2 Phaser diffractometer (Billerica, MA, USA), in reflection mode, at 35 KV and 40 mA, using nickel-filtered Cu-K radiation (1.5418 Å). Thermogravimetric analysis (TGA) of the ashes was performed using a TGA TA instrument SDT Q600 (TA Instrument, New Castle, DE, USA). About 10 mg of powder samples was heated in an alumina holder under nitrogen atmosphere from 20 to 800 °C at a heating rate of 10 °C/min. The morphology of the ashes was investigated by scanning electronic microscopy (SEM) using a Zeiss Scanning Electron Microscope Evo40. Energy dispersive X-ray (EDX) analysis was performed with a Bruker, XFlash detector 5010 [22,23].

### 2.2. Preparation of Thermoplastic Carbon Ashes/Maize Starch Films

**Figure 1:**
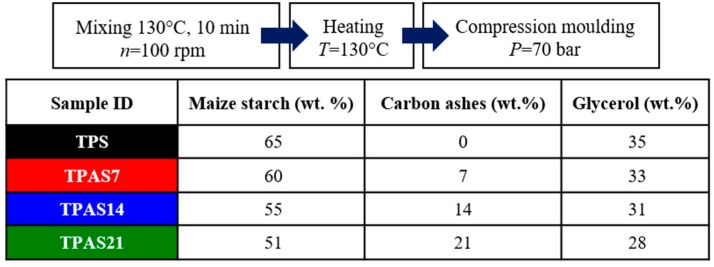
Production steps and settings of the thermoplastic starch films containing maize starch, glycerol and carbon ashes in different proportion. Weight composition of starch/carbon ashes samples.

The thermoplastic carbon ashes/maize starch (TPAS) films were manually prepared by pre-mixing maize starch neat powders with 35 wt.% of glycerol, respect to the starch matrix and recycled carbon ashes in an amount of 10 phr, 20 phr and 30 phr that correspond to 7 wt.%, 14 wt.% and 21 wt.% [17,18,19,20,21] (see Figure 1). The compounds were mixed at 130 °C for 10 min by using a Haake PolyLab System Rheomix 600/610 mixer (n = 100 rpm) (Haake, Karlsruhe, Germany) and then put in a Carbolite LHT6/120 oven (Carbolite, Derbyshire, UK) until they reached a temperature of 130 °C. Finally, they were compression molded, by means of a DGTS P7/91 press (DGTS, Veduggio (MB), Italy), at a pressure of 70 bar. Figure 1 reports the process parameters adopted, along with the weight composition and sample ID of each film produced.

### 2.3. Rheological Characterization

The effectiveness of glycerol as plasticizer was assessed by rheological analysis, using a TA Instrument Ares rheometer (TA Instrument, New Castle, DE, USA). Three tests were performed on starch plastisols with 35 wt.% of glycerol, by using a double plate geometry, setting a gap of 0.3 mm, a constant oscillatory amplitude (1%) and frequency (1 Hz), while increasing the temperature from 20 °C to 200 °C at 3 °C/min. An increase of the viscosity upon heating, due to the gelation process, was considered to be indicative of the plasticizing effectiveness of the glycerol. 

The effect of the different amounts of carbon-based ashes on the thermal and physical-mechanical properties of the molded thermoplastic carbon-based ashes/starch samples was assessed by using several techniques as following reported.

### 2.4. Wide Angle X-ray Diffraction

The film crystallinity was studied via WAXD analysis, on a Philips PW 1729 (Phillips, Eindhoven, the Netherlands), in reflection mode, using nickel-filtered Cu-Kα radiation (1.5418 Å). Hence, the degree of crystallinity of each film was calculated using the formula proposed by Hermans and Weidinger, as follows (Equation (1)) [24]:

(1) $$\%Crystallinity=\frac{Q_{st}-Q_{am}}{Q_{st}}\times 100,$$

where *Q_st_* and *Q_am_* are the areas calculated under the X-ray curves from the semi-crystalline and amorphous samples, respectively. 

### 2.5. Morphological Characterization

A Zeiss SUPRA 40 field emission scanning electron microscope (FESEM) was utilized to investigate the morphology of composite films. Images were acquired with an Everhart-Thornley detector at a working distance in the range 2.5–3 mm, electron acceleration voltage (extra-high tension, ETH) of 3.00 kV, magnification in the range 1.00–100.00 KX. Before SEM observations the samples were sputter-coated with 30 nm of Cr using a turbo-pumped sputter coater (Quorum Technologies, model Q150T). 

### 2.6. Differential Scanning Calorimetry

The glass transition temperature (*T_g_*) and melting heat (Δ*H_m_*) were estimated using a DSC, supplied by Mettler Toledo 622 (Mettler Toledo, Columbus, OH, USA). Dynamic DSC scans were performed from 20 to 250 °C, at a heating rate of 20 °C/min under nitrogen atmosphere. At least three measurements were performed on each sample. The value of the *T_g_* was calculated by identifying the point corresponding to the presence of an inflection (inflection point method). Thus, the *T_g_* coincides with the point at which the second derivative is zero.

### 2.7. Thermogravimetric Analysis

Thermogravimetric analysis is one of the most appropriate methods for the study of the thermal degradation and stability of polymeric blends as a key point of the optimization is indeed involved in the analysis of the decomposition of the material when subjected to heat increase. A first output given by the TGA curve consists in the threshold decomposition temperature, which allows identifying a process window for the polymer application. On the other hand, the threshold temperature of a polymeric blend could be not clearly identifiable if an overlapping of different phenomena, such as evaporation and degradation, occurs during the test. Therefore, in this work a study of the kinetics of the different decomposition processes was performed, in order to identify the degradation mechanism occurring during the degradation process. 

In particular, the thermal stability of the films was assessed by TGA, with a TA Instrument SDT Q600 (TA Instrument, New Castle, DE, USA). The samples were heated in an alumina holder, from 20 to 700 °C at a heating rate of 10 °C/min under nitrogen atmosphere; and three measurements were performed on each film. 

Additionally, the kinetic analysis of the TGA results [25] was carried out in order to evaluate the difference in thermal decomposition of starch systems before and after ash addition. 

Specifically, given a homogeneous system, the thermal decomposition can be expressed as:

(2) $$\frac{d\alpha \left(t\right)}{dt}=K\left(T\right)f\left[\alpha \left(t\right)\right],$$

where *K*(*T*) is the rate coefficient, which follows the Arrhenius equation, *α*(*t*) is the reaction extent of the component of the sample during degradation at a given time *t*, defined as:

(3) $$\alpha \left(t\right)=\frac{\left(w_{0}-w\left(t\right)\right)}{\left(w_{0}-w_{\infty }\right)},$$

with *w*_0_, *w*(*t*) and *w*_∞_ the weight of the sample before degradation, during degradation at a certain time t and after degradation, respectively.

The most commonly form for the differential conversion function, *f*(*α*(*t*)) is

(4) $$f\left(\alpha \left(t\right)\right)={\left(1-\alpha \left(t\right)\right)}^{n},$$

where *n* is the reaction order, assumed to remain constant during reaction [26].

Being *β* the heating rate, defined as *β* = *dT*/*dt*, Equation (2) can be expressed as follows:

(5) $$\frac{d\alpha \left(t\right)}{{\left(1-\alpha \left(t\right)\right)}^{n}}=\frac{A}{\beta }exp\left(-\frac{E_{a}}{RT}\right)dT,$$

where

- *E_a_* is the activation energy;
- *A* is the Arrhenius pre-exponential factor;
- *R* is the gas constant (8.31 J/mol·K).

Since degradation of starch can be assumed to be of first order in sample weight reaction [27] (i.e., n = 1), Equation (5) can be written as:

(6) $$ln\left(1-\alpha \left(t\right)\right)=-\frac{A}{\beta }\int_{T_{0}}^{T}exp\left(-\frac{E_{a}}{RT^{\prime }}\right)dT^{\prime }.$$

Since Equation (5) cannot be integrated in an exact form, the Broido method can be applied [28]; it assumes that the temperature range of analysis is close to the melting temperature and therefore (Equation (6)) that

(7) $$exp\left(-\frac{E_{a}}{RT}\right)≅{\left(\frac{T_{m}}{T}\right)}^{2}exp\left(-\frac{E_{a}}{RT}\right).$$

Equation (5) can be rewritten as:

(8) $$ln\left(1-\alpha \left(t\right)\right)=-\frac{ART_{m}{}^{2}}{\beta E}exp\left(-\frac{E_{a}}{RT}\right)$$

or

(9) $$ln(-ln(\left(1-\alpha \left(t\right)\right))=-\frac{E_{a}}{RT}+C.$$

By applying this method to the TGA analysis on starch samples, a straight line will be observed between ln(−ln((1−α(t))) and 1/T, with a slope of $-\frac{E_{a}}{R}$. Hence, by interpolating with a linear fit, the activation energy of the degradation process for each mixture can be calculated.

### 2.8. Moisture Absorption and Biodegradation Tests

The moisture absorption of the films was tested in a Binder KBF 115 controlled climate chamber (Binder, Tuttlingen, Germania), as a function of time at relative humidity (u.r.) of 75% and temperature of 23 °C. The tests were performed on three specimens of each film of rectangular shape kept at relative humidity of 50% and temperature of 23 °C, for three days long before the experiments. Each sample was periodically weighed, until a constant mass was achieved. The imbibition coefficient, *c_im_*, was calculated as (Equation (10)):

(10) $$c_{im}=\frac{w_{t}-w_{0}}{w_{0}}\times 100,$$

where *w_t_* is the mass of the samples at time, *t* = 48 h and *w*_0_ is the mass of the samples at time, *t* = 0. Biodegradation was tested on three rectangular (20 × 10 mm^2^) specimens. The test specimens were immersed in water, in sealed vials and placed in an oven for 312 h at 50 °C. The weight of each specimen was measured after 2, 4, 6, 24, 48, 144, 168, 192, 216 and 312 h.

### 2.9. Mechanical Characterization

Finally, the TPAS films containing 7 wt.% of ashes, were selected and characterized by tensile tests. The mechanical properties, such as the tensile strength, *σ_max_*, elastic modulus, *E* and ultimate strain, *ε_u_*, were measured at 22 °C, according to the ASTM D882-97 [29] (specimen dimension—120 × 12 × 0.4–0.6 mm) and by using a Lloyd LR5 K dynamometer (Lloyd Instruments Ltd., Bognor Regis, West Sussex, UK). The results were then compared to those of the TPS film (0% carbon ashes content). Five tests were performed on each film. The TPS and TPAS7 films were also mechanically characterized after moisture absorption, in order to assess the influence of humidity on the mechanical properties.

## 3. Data Processing and Experimental Results

### 3.1. Starch and Carbon Ashes Characterization

The structural, morphological and thermal characterization of the commercial Maizena starch was performed in a previous work [30]. The WAXD spectrum of the maize starch powder showed seven characteristic peaks and a degree of crystallinity of about 23.3%. The scanning electron microscopy (SEM) analysis evidenced the presence of circular granules. The DSC analysis showed a melting point of 120 °C and a melting heat of 41.5 J·g^−1^. Finally, the TGA curve exhibited a first degradation step from 50 to 95 °C, attributed to the water loss and a second step from about 296 to 322 °C, associated to the degradation of the starch [30].

The characterization of the milled ball carbon ashes was performed by WAXD and TGA in Reference [22]. The WAXD curve of the ashes (not shown here) exhibited several crystalline peaks which could be attributed to potassium chloride (KCl), sodium chloride (NaCl) and calcium carbonate (CaCO_3_) and only one peak at 2θ = 26.5°, which was related to graphite [22].

**Figure 2:**
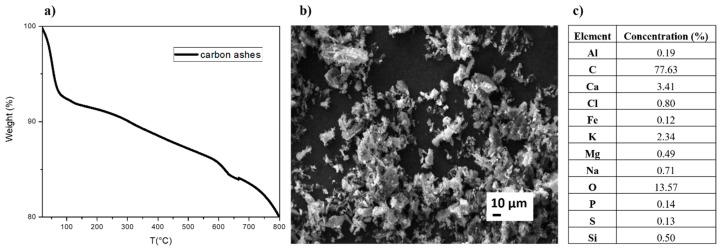
(**a**) Thermogravimetric analysis (TGA); (**b**) Scanning electron microscopy (SEM) image and (**c**) chemical composition by energy dispersive X-ray (EDX) of the ashes.

The TGA curves (reported in Figure 2a) entailed several weight losses steps due to the variety of carbon ashes composition. In details, the main steps observed are—the first step, ranging from room temperature to about 100 °C, attributed to the removal of the molecularly adsorbed water, the second step between 100 °C and 250 °C, originated from the removal of the thermally labile oxygen-containing functional groups [22] and the step ranging from 600° to about 700 °C due to the decomposition of CaCO_3_.

The SEM image of the ashes in Figure 2b reveals that the size of the fragments/particles is around 20 µm. The presence of NaCl, CaCO_3_ and KCl was also confirmed by SEM-EDX analysis as reported in the table inserted in Figure 2c. The most abundant element is carbon, present in a percentage of about 78%. The most plentiful elements, beside carbon, (>1%) are O, Ca and K. The following elements are present in an amount lower than 1%—Al, Cl, Fe, Mg, Na, P and S.

### 3.2. Thermoplastic Carbon Ashes/Maize Starch Films Characterization

#### 3.2.1. Rheology

Rheological analyses were performed on starch plastisols mixed with 35 wt.% of glycerol, at room temperature (TPS film composition). Previous works were carried out with the aim of optimizing the type and the amount of plasticizer, thus leading to the conclusion that the addition of 35 wt.% of glycerol involves the highest plasticization yields [30]. 

**Figure 3:**
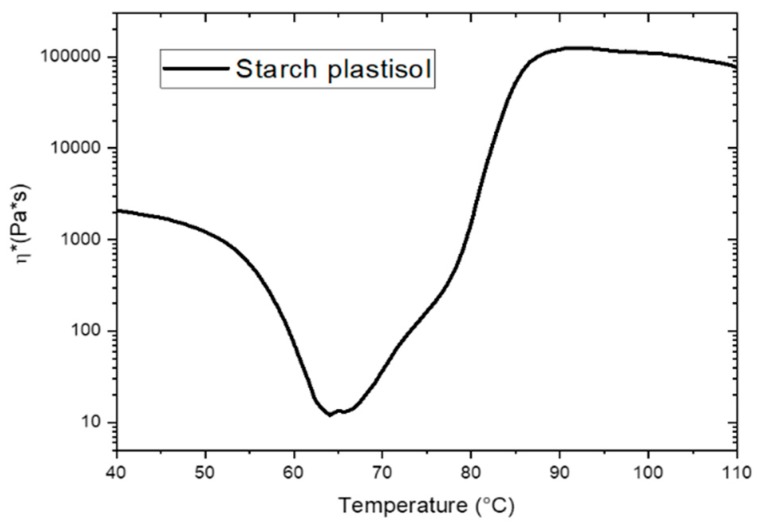
Rheological analysis on starch plastisol with 35 wt.% of glycerol.

The analysis of the complex viscosity during heating provides information about the evolution of the gelation process, which involves adsorption of the plasticizer by the starch powders. As observed in Figure 3, the gelation process involves a significant increase of the viscosity of the plastisol between 70 °C and 85 °C. Such increase is attributed to the swelling of the starch particles due to glycerol absorption [31] and therefore plasticization of the polymer. First, the viscosity decreases as a result of heating. The plastisol is a suspension, with the starch particles dispersed within the plasticizer. Afterwards, at a sufficiently high temperature (depending on the type of plasticizer, on the characteristics of the polymer and on the concentration of plasticizer) the plasticizer is physically absorbed by the starch particles with their consequent swelling. The particles grow in size and touch themselves, with a resulting increase in the viscosity of the compound. Finally, a second increase in viscosity occurs, due to the swelling of the starch particles with higher dimensions. The analysis of the dynamic viscosity of the component therefore provides important information regarding the process window; the viscosity curve of TPS in Figure 3 shows a final swelling temperature of 90 °C, therefore, a process temperature higher than 90 °C must be chosen in order to ensure a complete swelling of the material during the mixing phase. 

The mixing temperature was chosen also taking into account the melting temperature of starch; as shown in Figure 6a, TPAS melting starts at 125 °C, therefore, processing starch and glycerol at 130 °C allows a partial melting of the components and improves their mixing.

Since the plasticization involves only the polymer and is not affected by the presence of the ashes, the same swelling conditions were found for every amount of carbon ashes added to the plastisol.

#### 3.2.2. Morphology

In order to analyze the crystalline structure of the TPAS films, those were characterized by XRD analysis and the results were compared to that of the thermoplastic maize starch film (TPS).

**Figure 4:**
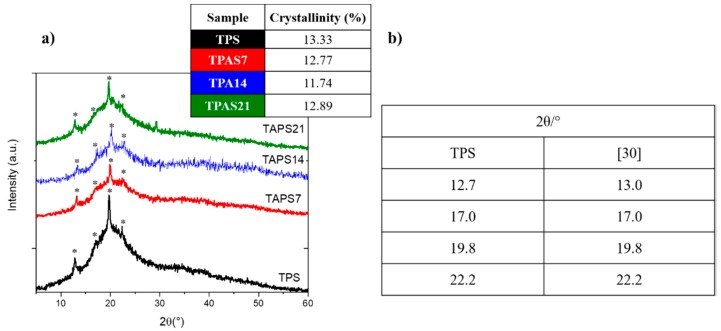
(**a**) X-ray diffraction (XRD) spectra of the thermoplastic recycled carbon ashes/starch (TPAS) films compared to that of the thermoplastic maize starch film and crystallinity values in the insert; (**b**) characteristic peaks of the TPAS films obtained by XRD curves compared to the literature data [30] on thermoplastic starch films [32].

The results confirm the semi-crystalline nature of all starch films, both in presence than in absence of ashes (Figure 4a). In particular, all the prepared TPAS samples show a first peak at 2θ = 12.7°, a second peak at 2θ = 17.0°, a third one at 2θ = 19.8° and the last one at 2θ = 22.2° (Figure 4b). The findings are comparable to the literature data on the thermoplastic starch films [32]. Therefore, the presence of the filler does not influence the crystal planes of the starch, as all the peaks appear at the same 2θ angles. 

The XRD diffraction patterns of all samples were used to estimate the degree of crystallinity, as the ratio between the area of the peaks and the total area of the XRD pattern, including the amorphous halo band (Equation (1)). Results, reported in the insert of Figure 4a, indicate that the filler amount does not affect the crystallinity of starch, since the same crystalline fraction is present in 7 wt.% and 21 wt.% c ashes embedding films. 

**Figure 5:**
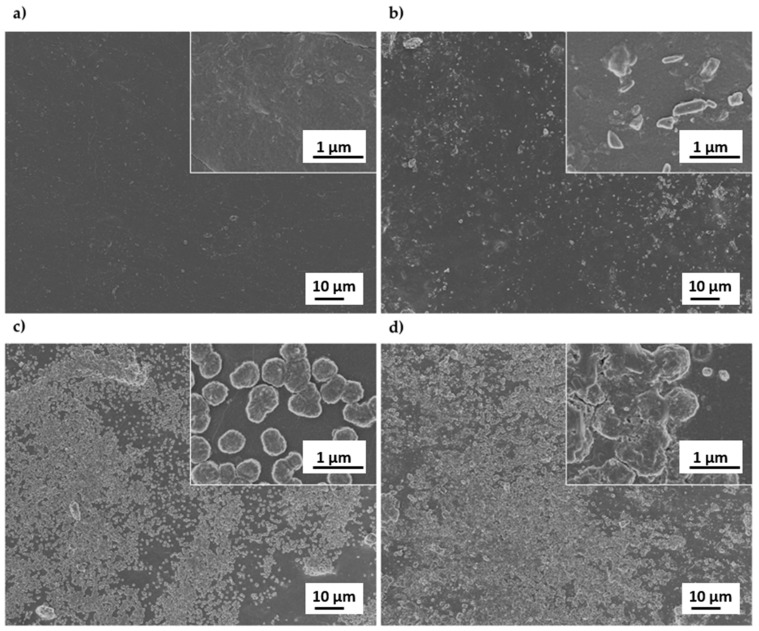
Representative SEM images of (**a**) TPS; (**b**) TPAS7; (**c**) TPAS14 and (**d**) TPAS21 films at lower (1 KX) and higher (100 KX) magnification.

Representative SEM images of TPS and TPAS films are shown in Figure 5. The low-magnification images demonstrate the presence of increasing ashes concentration between TPAS7 and TPAS21 films. Particularly, a good carbon ashes dispersion can be observed for TPAS7, while an inhomogeneous distribution with the formation of aggregates and clusters is observed in TPAS14 and, especially, in TPAS21 as evidenced by high-magnification SEM images. 

#### 3.2.3. Thermal Stability

**Figure 6:**
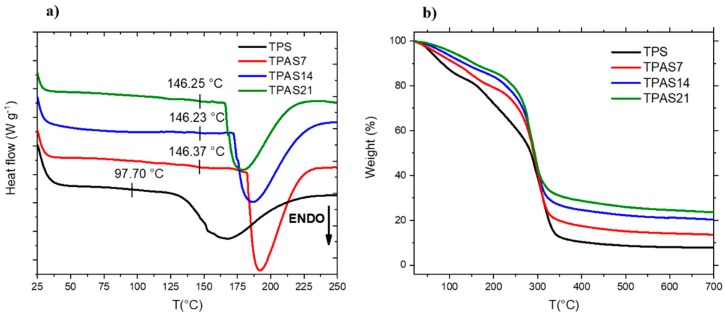
(**a**) Differential scanning calorimetry (DSC) and (**b**) TGA spectra of the thermoplastic recycled carbon ashes/starch (TPAS) films compared to that of the thermoplastic maize starch film.

Figure 6a shows the DSC curves of each film, evidencing the presence of the glass transition temperature and an endothermic peak. Several authors investigated the nature of the endothermic peak. Hanna et al. [33] attributed the endothermic peak to a residual gelation of the starch. Nevertheless, as shown in Figure 3, this observation is inconsistent with rheological findings, since the swelling process occurs at lower temperatures and it is independent from the presence of carbon ashes. Other authors [34,35] attributed the endothermic peak to both melting and thermal decomposition of the starch but this last hypothesis is not consistent with TGA results (Figure 6b), which, as will be discussed later, show a degradation temperature higher than that of the endothermic peak. Therefore, according to the authors in References [34,35] the endothermic peak found in Figure 6a can be ascribed only to the melting of the starch crystals. The glass transition temperature (*T_g_*), the melting point (*T_m_*) and the melting enthalpy (Δ*H_m_*) of each film were calculated from the curves of Figure 6a. In particular, the glass transition was calculated by identifying the point corresponding to the presence of an inflection and the melting enthalpy was calculated by normalizing the weight of each sample with the fraction of starch.

Table 1 highlights a strong influence of carbon ashes on the *T_g_* of the system. In particular, the presence of the filler involves a change in the chain mobility of the starch amorphous phase, thus leading to an increase of the glassy region, with a *T_g_* shift of about 50 °C, regardless the amount of the ashes.

The presence of fillers involves a raise in glass transition temperature as the filler dispersion into the polymer chains causes a decrease in its mobility. The glass transition temperature of the polymer increases with increasing filler concentration and with increasing specific surface area of the filler. In addition, this increase is influenced by the distribution of the filler in the amorphous phase of polymer [36]; starting from these considerations, it should be expected that the glass transition temperature increases with increasing ashes content. On the other hand, high quantities of ashes are also leading to clusters formation (Figure 5), for these reasons comparable glass transition temperatures were recorded for every amount of filler.

The addition of carbon ashes also involves an alteration of the starch crystallization process. DSC curves of TPAS show indeed a shift in the melting peak temperature, principally due to an increase in crystallite size, rather than a modification of the overall crystallinity [37]. Specifically, Table 1 shows a decrease of the melting temperature as the ashes content is raised. This result is adducible again to the dispersion homogeneity of the filler, being sample TPAS7 the most uniform composite it alters the crystallization TPS process at most, (development of thicker crystals). The melting enthalpy of TPS, which is correlated to the crystalline fraction, instead, seems not to be strongly affected by the presence of the ashes; and only a slight decrease is detected in presence of ashes. The results are in agreement with the XRD data, where the use of carbon ashes was found not to considerably modify the crystalline fraction of the thermoplastic starch films and composites.

Thermogravimetric analyses were performed on all the sample produced, in order to evaluate the effect of the ashes on the starch thermal stability. The solid residue and the water weight loss are deduced from Figure 6b and they are also reported in Table 1. All the samples show a first weight loss below 150 °C, mainly attributed to the water evaporation and a second loss between 150 °C and 350 °C, due to the contemporary evaporation of glycerol and the degradation of starch. In particular, when compared to TPS, the water content decreases by about 20%, 22% and 38% with a carbon ashes content of 7 wt.%, 14 wt.% and 21 wt.%, respectively. As a such, the presence of ashes reduces the water absorption of the starch. Due to the overlap of the glycerol evaporation and starch degradation phenomena, taking place in the range of 150 °C and 350 °C, no clear identification of the starch degradation temperature was possible. Therefore, since the evaporation of glycerol occurs up to 280 °C, a kinetic analysis was carried out in a range of temperature between 280 °C and 320 °C, to study the difference in the degradation rate with and without ashes addition. The step ranging from 600° to about 700 °C, associated to the decomposition of CaCO_3_, in the carbon ashes curve (see Figure 2a) is not evident in the composites curves, maybe due to the sensibility of the instrument respect to the low CaCO_3_ concentration. 

**Figure 7:**
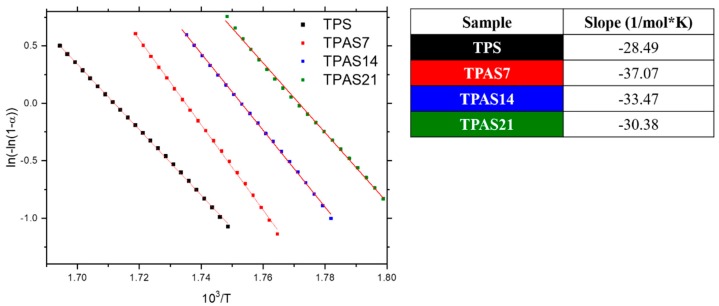
ln(-ln(1-α)) vs. 1/T plot using Broido method for the thermoplastic starch film (TPS) and thermoplastic recycled carbon ashes/starch films (TPAS) with different ashes contents.

Figure 7 reports the results of the Broido analyses [28] applied to TPS and TPAS samples with different ashes contents. An insert in Figure 7 shows the activation energy *E_a_*, calculated from the slope of each curve, with a linear fit of the data based on Equation8 (see material and methods). Against to TPS, the data show an increase of 30%, 20% and 10% in activation energy for the TPAS7, TPAS14 and TPAS21 samples, respectively, with the best result achieved for the lowest amount of carbon ashes added. The increase in activation energy is then an index of the higher thermal stability caused by presence of the ashes and their dispersion quality. 

**Figure 8:**
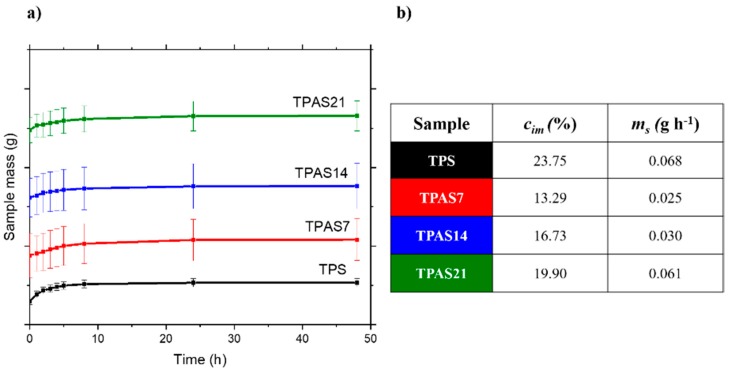
(**a**) Moisture absorption; (**b**) imbibition coefficient and rate of absorption of the thermoplastic starch (TPS) and thermoplastic recycled carbon ashes/starch (TPAS) films.

The moisture absorption capacity of the thermoplastic films was assessed in extreme conditions of humidity (75% u.r.) (see materials and methods). Figure 8a shows the average mass calculated from three samples for each composition and the respective standard deviation as a function of the time, while Figure 8b reports the imbibition coefficient, *c_im_*, calculated by Equation (2) and the rate of absorption, calculated from the slope, *m_s_*, of the initial portion of the curves for each film. The imbibition coefficient of the TPS film decreases from 23.75% to 13.29% (i.e., 44%), from 23.75% to 16.73% (i.e., 30%) and from 23.75% to 19.90% (i.e., 16%) when 7 wt.%, 14 wt.% and 21 wt.% of ashes are added, respectively. The rate of absorption also is 0.068, 0.025, 0.030 and 0.061 g/h for TPS, TPAS7, TPAS14 and TPAS21 films, respectively. Therefore, the TPAS7 not only absorbs less water than TPS, TPAS14 and TPAS21 but it does it more slowly. This latter behavior could be attributed to the homogenous distribution of the carbon ashes into the polymeric matrix. The ashes, in fact, do not contribute to the water absorption of the starch film and, above all, they probably create a “network“ among the starch molecules, similar to a tortuous path towards the water molecules, hindering, thus the swelling, due to the water absorption.

**Figure 9:**
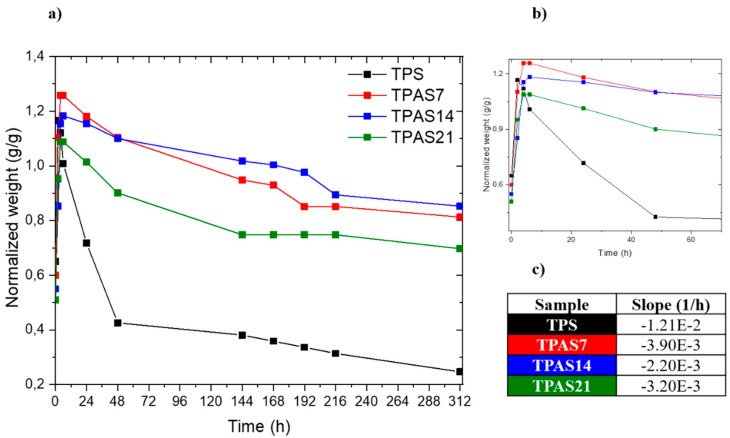
(**a**) Degradation curves of the thermoplastic starch (TPS) and thermoplastic recycled carbon ashes/starch (TPAS) films in terms of weight as a function of time; (**b**) zoom of the degradation curves from 0 to 70 h; (**c**) slope values of each degradation curve.

The biodegradability of the produced films was also investigated, in order to assess the influence of carbon ashes on the hydrophilicity of the starch. The weight loss was calculated and normalized by considering only the starch amount present in every sample. As shown on the left in Figure 9, for all samples a sharp weight loss occurs in the first 48 h, followed by a lower degradation rate for longer times. The dissolution rate was then evaluated by the linear fit of the slope for each curve in the first 48 h of test, zooming the curve from 0 to 70 h (see insert on the right in Figure 9). The table in Figure 9 also reports the slope values of each curve. The presence of ashes was found to strongly decrease the deterioration of starch, independently of the filler amount; as shown in Figure 9, an average reduction of 70% in the dissolution rate was reached with the addition of carbon ashes.

#### 3.2.4. Mechanical Analysis

The effect of carbon ashes on TPS mechanical response was investigated through tensile tests, according to ASTM D882-97 [29]. In particular, based on results shown above, the addition of 7 wt.% of carbon ashes involved better results in terms of filler distribution and thermal stability. Therefore, tensile tests were performed only on TPAS7 films and compared with TPS samples.

**Figure 10:**
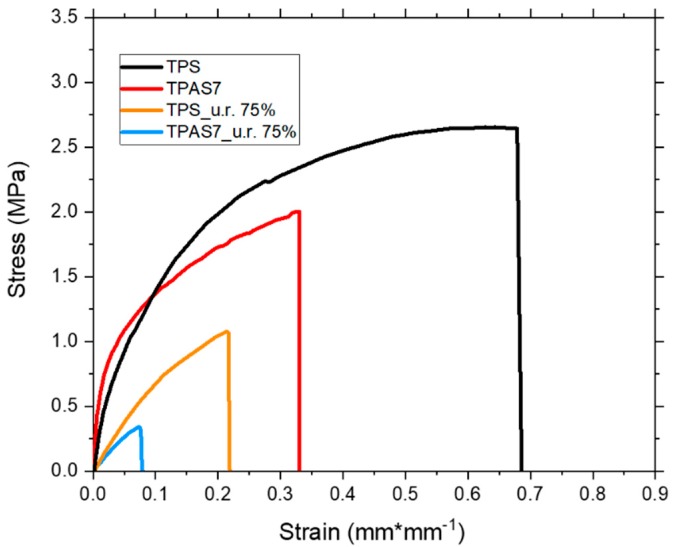
Stress-strain curve of the thermoplastic recycled carbon ashes/starch sample with an amount of 7 wt.% of ashes (TPAS7) in comparison to that of the thermoplastic starch film (TPS), before and after the weathering tests.

The results, reported in Figure 10, show a slight stiffening effect of the filler, with an increase of about 20% in tensile modulus. On the other hand, the addition of carbon ashes involved a decrease of both tensile strength and elongation at break (Table 2), due to the increase in defects caused by the addition of the filler. In fact, though an amount of 7 wt.% of carbon ashes was found to ensure a better distribution (Figure 5), the presence of clusters and imperfections cannot be excluded. In this sense better mechanical properties can be reached by improving ashes distribution.

The moisture ageing caused, in any case, a reduction of the tensile modulus of the samples, since the increased water content acted as plasticizer for both TPS and TPAS7 films, after the weathering tests (samples named TPS_u.r. 75% and TPAS7_u.r. 75%, respectively). On the other hand, a decay in mechanical response was detected, with a sharp decrease of tensile strength and deformation at break for all the tested samples; this result can be attributable to a partial dissolution of the starch after the water exposure during moisture ageing.

## 4. Conclusions

In this work, an innovative approach for the recycling of carbon waste ashes by producing bio-composite thermoplastic starch films for agricultural applications was proposed. 

Several TPAS films were prepared by hand pre-mixing neat maize starch powder and 35 wt.% glycerol in presence of different amounts of waste carbon ashes ranging from 7 wt.% to 21 wt.%. The compounds were mixed at 130 °C for 10 min and then compression molded. The effect of the different amounts of carbon ashes on the thermal and physical-mechanical properties of TPAS samples was assessed by using several techniques. 

The XRD diffraction of all films produced showed that the presence of the carbon ashes does not influence the crystal planes of the starch. Moreover, the crystallinity of starch seems not to be directly affected by the presence of the filler. The SEM analysis indicated a good dispersion of the ashes for TPAS7 and a higher inhomogeneity in the distribution of the filler, besides the presence of carbon ashes clusters, for TPAS14 and, especially for TPAS21.

The DSC studies of each film evidenced the presence of an endothermic inflection point, related to the glass transition temperature and an endothermic peak, which can be ascribed to the melting of the starch crystals. The presence of the filler determined a *T_g_* shift of about 50 °C, independently from the amount of the ashes. Furthermore, the increase in melting temperature in presence of ashes was ascribed to a change in the crystallization process, with a consequent increase in the crystallite dimension. On the other hand, the melting enthalpy of TPS seemed not to be strongly affected by the presence of the ashes.

The TGA analysis showed a reduction of the water absorption in presence of the starch, with a decrease in water content of about 20%, 22% and 38% with a carbon ash amount of 7 wt.%, 14 wt.% and 21 wt.%, respectively. These results were confirmed by the moisture absorption tests which indicated that the imbibition coefficient of the TPS film decreases by about 44%, 30% and 16% when 7 wt.%, 14 wt.% and 21 wt.% of ashes are added. 

A kinetic analysis was carried out on a selected range of temperature (from 280 to 320 °C), where only degradation of the starch occurs, to study the difference in the degradation rate with and without ashes addition. Compared to TPS, the activation energy increases of 30%, 20% and 10% for TPAS7, TPAS14 and TPAS21, respectively. This increase in activation energy is an index of a higher thermal stability caused by the addition of the ashes.

The presence of ashes also decreases of 70% the deterioration of the starch, independently of the filler amount.

Finally, the tensile tests showed a slight stiffening effect of the filler, with an increase of about 20% in tensile modulus, for the TPAS7 film.

## Figures and Tables

**Table 1:** DSC and TGA values of thermoplastic starch (TPS) and thermoplastic recycled carbon ashes/maize starch (TPAS) films.

Sample	*T_g_* (°C)	*T_PEAK_* (°C)	Δ*H_m_* (J·g^−1^)	Water Loss (%)	Solid Residue (%)
TPS	97.70	167.51	219.14 ± 1.30	14.96 ± 1.87	7.74 ± 0.07
TPAS7	146.37	191.95	217.73 ± 0.85	12.03 ± 0.86	13.62 ± 0.13
TPAS14	146.23	187.18	212.51 ± 2.00	11.54 ± 0.61	20.30 ± 0.19
TPAS21	146.25	179.48	217.52 ± 3.25	9.30 ± 0.53	23.55 ± 0.11

**Table 2:** Mechanical properties of the thermoplastic starch film (TPS) and thermoplastic recycled carbon ashes/starch film with an amount of 7 wt.% of ashes (TPAS7) before and after weathering tests.

Sample	*σ_f_* (MPa)	*ε_u_* (mm * mm^−1^)	*E* (MPa)
TPS	2.65 ± 0.03	0.66 ± 0.05	27.87 ± 0.91
TPS_u.r. 75%	1.08 ± 0.04	0.23 ± 0.01	8.07 ± 0.51
TPAS7	2.27 ± 0.16	0.33 ± 0.03	32.19 ± 0.66
TPAS7_u.r. 75%	0.52 ± 0.10	0.07 ± 0.02	5.86 ± 0.49
